# A reproducible workflow for 3D finite-element modeling of strike-slip duplex deformation in Abaqus

**DOI:** 10.1016/j.mex.2026.103917

**Published:** 2026-04-19

**Authors:** Anis Khalifeh-Soltani, Seyed Ahmad Alavi, Reza Derakhshani

**Affiliations:** a Department of Oil and Sedimentary Basins, Faculty of Earth Sciences, Shahid Beheshti University, Tehran, Iran; b Department of Geology, Shahid Bahonar University of Kerman, Kerman, Iran; c Department of Earth Sciences, Utrecht University, Utrecht, the Netherlands

**Keywords:** Strike-slip fault, Duplex structures, Convergence angle, Transpressional tectonics, Transtensional tectonics, Fault mechanics, Structural geology

## Abstract

This article presents a fully reproducible workflow for three-dimensional finite-element modeling of deformation in contractional and extensional strike-slip duplexes subjected to variable convergence angles. The method is implemented in Abaqus and provides a standardized procedure for constructing model geometries, assigning elastic–plastic rheology, defining frictional fault interactions, and applying displacement boundary conditions representative of oblique strike-slip motion. The workflow enables systematic variation of the convergence angle while preserving geometric and mechanical similarity across models, allowing users to generate directly comparable stress, strain, and displacement fields. All model input data, output datasets, and visualization scripts are supplied in an open repository to ensure full transparency, reproducibility, and adaptability to other tectonic settings. This methodological framework can be readily integrated into broader geomechanical, structural, or seismotectonic investigations requiring controlled numerical experiments on strike-slip fault systems.

• Provides a standardized and reproducible 3D finite-element workflow for modeling deformation in strike-slip duplex systems.
• Enables systematic variation of convergence angle while maintaining consistent geometry, rheology, and boundary conditions.
• Includes complete Abaqus input data, documentation, and visualization resources for direct replication and adaptation.

## Graphical abstract

**Figure:**
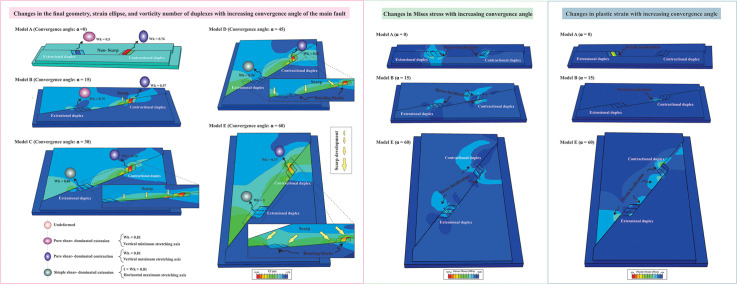

## Specifications table

**Table:** 

**Subject area**	Earth and Planetary Sciences
**More specific subject area**	Tectonics, Fault Mechanics, Finite Element Modeling
**Name of your method**	Abaqus-based finite-element modeling workflow for strike-slip duplexes
**Resource availability**	Data available in Mendeley Data repository [1]

Related research article

Khalifeh-Soltani, A., Alavi, S. A., & Derakhshani, R. (2026). Evaluating the role of convergence angle in the geometry of contractional and extensional strike-slip duplexes using 3D finite element models. Journal of Structural Geology, 202: 105570 [2].

## Background

Understanding the mechanical behavior of strike-slip fault systems requires numerical methods capable of capturing complex three-dimensional deformation. Among the structural configurations observed along major strike-slip faults [2] contractional and extensional duplexes represent key zones of deformation partitioning, local strain accumulation, and structural complexity [3,4]. Their internal deformation patterns are influenced by several geometric and kinematic parameters, including the orientation of the main fault relative to the applied displacement field [5,6]. Investigating these systems therefore requires a modeling framework that can systematically vary these parameters while maintaining control over material properties, boundary conditions, and geometric configurations.

Finite-element modeling provides a powerful tool for analyzing stress and strain distributions within fault-related structures [7,8], including recent studies that quantify shear stress evolution in fault-related folds and their implications for subsurface geomechanics (e.g. [9]). However, reproducibility in such modeling depends strongly on transparent reporting of model geometry, material behavior, mesh configuration, and boundary conditions [10]. Many existing studies in structural geology describe model results without publishing the full methodological workflow, which makes independent replication challenging. Models implemented in commercial FE software such as Abaqus [11] are often described only briefly, and key implementation steps—such as volume construction, assignment of fault interfaces, and application of oblique convergence—are seldom presented in a level of detail sufficient for reuse by other researchers.

The present methodology was developed to ensure that the construction, implementation, and execution of three-dimensional finite-element models of strike-slip duplexes can be replicated reliably. It provides step-by-step procedures for developing consistent model geometries, defining elastic–plastic rheological parameters, assigning displacement boundary conditions, and extracting stress–strain relevant to fault analysis. In addition to the descriptive workflow, complete input data used in the simulations are provided in an open repository. These data allow users to reproduce the models directly or adapt them to alternative tectonic settings, duplex configurations, or material properties.

The motivation for publishing this workflow is twofold. First, the modeling procedure itself has broad applicability in studies of strike-slip tectonics, beyond the specific research questions addressed in the associated scientific article [2]. The same workflow can be adapted to investigate a wide range of deformation problems that involve distributed shear, block interactions, or oblique convergence. Second, making the methodology fully transparent and reusable aligns with current best practices in computational geosciences, particularly regarding accessibility of numerical models and reproducibility of results.

This article complements the associated research publication [2] by documenting the full computational procedure behind the finite-element models. Whereas the research article [2] focuses on the geological implications of varying the convergence angle across strike-slip duplexes, the present article describes the technical steps required to construct and run the models. By isolating the methodological content, this article enables other researchers to reuse, adapt, or extend the modeling framework for their own investigations. This includes applications in structural geology, tectonic modeling, fault mechanics, and geomechanical analysis of complex fault networks. In this study, the term “fault” or “fault system” is used to describe the modeled structures, as the internal components of natural fault zones (e.g., damage zones and fault cores) are not explicitly represented.

## Method details

This section provides a complete description of the workflow used to construct, parameterize, and execute three-dimensional finite element (FE) models of contractional and extensional strike-slip duplexes in Abaqus. The procedures are written to enable full reproducibility by other researchers and can be applied directly to new tectonic scenarios with minor modification of geometry or boundary conditions. Input data and output files of models, which include Excel tables, figures, and animations, are presented in the Mendeley Data repository [1].

• Building and running a model in Abaqus is done through its modules, which include:
• Part: Building model parts separately.
• Property: Definition of mechanical properties of model parts and stress-strain relationships.
• Assembly: Building a model by assembling its parts and determining the coordinate axes.
• Step: Definition of modeling steps, solution method, and outputs.
• Interaction: Defining the interaction of model parts.
• Load: Definition of boundary conditions and loading.
• Mesh: Defining the type and size of meshes.
• Job: Job creation and model running.
• Visualization: Displaying the final geometry and model outputs, and extracting them.

## Model architecture and geometric construction (part module)

### Model domain definition

The models consist of two volumes:

• Upper crustal block: 400 m (Z) × 2000 m (X) × 50 m (Y).
• Basement block: 400 m (Z) × 2200 m (X) × 60 m (Y).

The coordinate system follows the Abaqus convention:

• X – Model length.
• Z – Model width.
• Y – depth (positive upward).

The basement extends 100 m longer in the X-direction to accommodate strain shadowing at the model boundaries and to ensure that fault-parallel displacement does not interact with the lower boundary.

### Duplex geometry

Two duplex systems are embedded within the upper block:

• Extensional duplex.
• Contractional duplex.
• Each duplex consists of 5 blocks; the three middle blocks are cubic with a parallelogram base and the two marginal blocks are wedge-shaped.each measuring: Length: 100 m.
• Width: 60 m.
• Height: 50 m (entire upper block thickness).
• Fault dip: 60°.

The duplexes are separated by 520 m along strike.

Tips for construction:

• First, create the upper and lower blocks of the models.
• Sketch the geometry of the main fault and duplexes in the XY plane on the upper block of models by the partition tool.
• Partition the upper block using the extrude tool.

### Convergence angle

The convergence angle (α) is defined as the angle between:

• the strike of the main fault.
• and the imposed displacement vector (-X direction).

α is systematically varied: 0°, 15°, 30°, 45°, 60°.

A separate simple strike-slip model (Model F) is created by removing duplex partitions while preserving all boundary conditions, geometry, and rheology.

## Material properties and rheology (property module)

### Constitutive behavior

All models follow elastic–plastic material behavior:

• Elasticity: Hooke’s law.
• Plasticity: Mohr–Coulomb criterion.

### Mechanical parameters

**Table:** 

**Property**	**Value**
Density (ρ)	2400 kg/m³
Young’s modulus (E)	27 GPa
Poisson’s ratio (ν)	0.38
Cohesion (C₀)	13 MPa
Internal friction angle (φ)	26.5°
Dilatancy angle (ψ)	13.4°

These parameters are representative of consolidated sedimentary and crystalline upper-crustal materials and are based on previously published numerical and geomechanical studies. In particular, the parameter set is consistent with that used in the associated research article [2], ensuring direct comparability between the methodological workflow and the reported simulation results. The selected values of Young’s modulus, Poisson’s ratio, cohesion, and internal friction angle fall within commonly reported ranges for brittle upper-crustal rocks, as documented in rock mechanics and geodynamics literature, as well as in finite-element studies of fault-related deformation (e.g. [7,9,12]). The dilatancy angle (ψ) was selected as approximately half of the internal friction angle (φ), which is a commonly adopted assumption in Mohr–Coulomb plasticity models for geomaterials. This relationship reflects the tendency of brittle rocks to exhibit limited volumetric expansion during shear deformation compared to the maximum shear resistance defined by the friction angle and has been widely used in numerical simulations of upper-crustal deformation (e.g. [13,14]). Using the same set of mechanical parameters across all models ensures that the convergence angle remains the only variable controlling variations in deformation behavior.

## Assembling the model parts (assembly module)

Since the blocks were made with precise coordinates, it was easy to assemble the model by calling each part, they were called as independent parts.

## Definition of modeling steps (step module)

All models contain one step, and its procedure type is defined as static general.Increment control:

• Automatic type.
• Maximum number of increments: 1000.
• Maximum increment: 0.01.
• Minimum increment: 1E-010.

## Fault implementation (interaction module)

### Contact and interaction definition

All fault surfaces are assigned:

• Tangential behavior: penalty friction with μ = 0.01.
• Normal behavior: hard contact.

The low friction coefficient ensures shear localization along the prescribed slip surfaces and is consistent with previous numerical studies of strike-slip systems.

### Best practice

• Ensure all fault surfaces are defined before meshing.
• Validate normals: Abaqus is sensitive to surface orientation.
• Use “flip normal” cautiously; recheck interaction direction.

## Boundary conditions and loading (load module)

### Basement fixing

All basement nodes are pinned:

Ux = Uy = Uz = 0

This immobilizes the lower block and prevents rigid-body motion. This boundary condition represents a simplifying assumption that isolates deformation within the upper crustal block and ensures numerical stability by preventing rigid-body motion of the model domain. In natural strike-slip fault systems, the basement may also accommodate deformation; however, the present approach is designed to focus on the relative deformation patterns within the duplex structures rather than simulating fully coupled crustal behavior. As a result, the fixed basement condition may lead to an underestimation of deformation at depth, but it does not significantly affect the comparative analysis of stress, strain, and displacement patterns between models with different convergence angles. This simplification is consistent with the modeling strategy adopted in the associated study [2], ensuring comparability between the workflow and the reported results.

### Upper block constraints

Movement (Uz = 0) and rotation (UR3= 0) in the z direction are limited at the front and back boundaries of the upper blocks of the models. Movement (Ux= 0) and rotation (UR1= 0) in the X direction are limited to the left and right boundaries of the back upper block.

### Applied displacement

Dextral strike-slip deformation is imposed by applying a 20 m displacement in the -X direction is applied to the right boundary of the front upper block, while its left boundary is free. The magnitude of the applied displacement (20 m) is defined relative to the model dimensions and is selected to ensure sufficient deformation for analyzing stress, strain, and geometric evolution within the duplex structures. This value does not represent a specific natural displacement but is scaled to produce measurable and stable deformation patterns within the numerical model. Smaller displacements resulted in limited strain development, whereas larger displacements increased computational cost without significantly altering the overall deformation style. Therefore, 20 m represents a practical balance between numerical stability, computational efficiency, and the ability to capture the full evolution of deformation. This displacement magnitude is consistent with that used in the associated study [2], ensuring direct comparability between the methodological workflow and the reported simulation results.

## Meshing strategy (mesh module)

### Element shape and technique

Hexahedral elements and structured meshing techniques were avoided due to the geometric complexity introduced by multiple partitions and inclined fault planes within the duplex structures. These features hinder the generation of a structured hexahedral mesh without producing highly distorted elements or requiring extensive manual mesh control. Therefore, wedge elements (C3D6 in Abaqus) were used in combination with the sweep meshing technique, as they provide greater flexibility for discretizing irregular geometries while maintaining acceptable element quality. This approach is commonly adopted in finite-element models involving complex fault geometries where structured meshing is not feasible.

### Mesh size

A mesh-independence test was conducted to ensure that the numerical results are not significantly affected by element size. Three mesh resolutions with element sizes of 10 m, 20 m, and 30 m were tested. The results were compared in terms of displacement fields and strain distribution within the duplex structures, consistent with the modeling framework used in the related research article [2]. The comparison showed that the differences between the 20 m and 10 m meshes were negligible, whereas the 30 m mesh resulted in a reduced resolution of strain localization. Therefore, a uniform element size of 20 m was selected as an optimal compromise between computational efficiency and solution accuracy. This mesh resolution is also consistent with that used in the associated study, ensuring comparability between the methodological workflow and the reported scientific results.

## Model running (job module)

• A job is created to run the model.
• Data is checked before submit the model for running.

## View results and extract outputs (visualization module)

The geometric changes of the model during modeling can be viewed and extracted in image (e.g., PS, EPS, PNG, TiFF, SUG) and animation (AVI) format in this module.

Other outputs that are determined in the step module can also be extracted, these outputs include:

• Principal stresses and their components.
• Principal strains and their components (Both elastic and plastic strain).
• Displacement of elements and their components.

### Manual outputs

• Some outputs have also been measured manually by the Query tool, including:
• Fault-scarp relief.
• Duplex width change (ΔW).
• Duplex relief (ΔR).

### Results extracted from outputs

Automatic and manual outputs extracted from Abaqus can be used for other calculations and obtain new data, for example:

The strain ellipse has been calculated and plotted based on the changes in width (ΔW) and relief (ΔR) of the duplexes.

The kinematic vorticity number (Wk = *cos* α) is calculated based on the angle of the duplexes relative to the direction of displacement.

## Method validation and verification

Verification confirms a numerical model is the correct representation of the mathematical model, while validation confirms the model accurately represents the real world. Verification checks for coding and numerical accuracy, whereas validation compares the model's results to experimental or real-world data to ensure physical accuracy. The goal of verification is to ensure the numerical implementation correctly solves the underlying equations, while the goal of validation is to determine whether the model accurately represents real-world physics for its intended use.

The validation of the work has been done by comparing the results of the models with natural examples and concepts of strike-slip duplexes, which show a good agreement. Also, the verification has been done through mesh verify for all models, which shows that the errors and warnings across all are zero. models. All verification outputs are provided in the accompanying Mendeley Data repository [1].

1. Validation with natural examples

Although the primary purpose of this article is methodological, the deformation patterns generated by the workflow were checked against field-scale observations from:

• the Qom–Zefreh fault system (Iran).
• the Qir–Hurm strike-slip duplex (Iran).

Specifically, the spatial distribution of:

• uplift zones.
• subsidence regions.
• strain concentration.
• scarp offsets.

showed qualitative agreement with mapped duplex structures, validating that the numerical method reproduces realistic strain geometries when applied to natural fault geometry and structural configurations.

**2. Animation-based verification**

The repository includes AVI animations illustrating incremental deformation during model execution. These animations are used as a qualitative verification tool to assess the consistency and stability of the numerical results. The following criteria were systematically evaluated:

• Displacement continuity: No abrupt or unrealistic jumps in nodal displacement between increments.
• Numerical stability: Absence of oscillations, divergence, or irregular deformation patterns during the simulation.
• Strain evolution: Smooth and progressive accumulation of strain without artificial localization caused by numerical artifacts.
• Element behavior: No excessive distortion or collapse of elements throughout the deformation process.
• Kinematic consistency: Progressive and physically consistent rotation and displacement of duplex blocks in accordance with the imposed boundary conditions.

These criteria ensure that the numerical solution evolves in a stable and physically meaningful manner throughout the simulation. This qualitative verification complements the mesh verification and supports the reliability of the modeling workflow.

## Limitations

The workflow presented here is robust, but several limitations should be considered. The models use predefined fault geometries and therefore cannot simulate spontaneous fracture initiation or fault propagation. The elastic–plastic Mohr–Coulomb rheology captures upper-crustal behavior but does not include viscoelastic, thermal, or rate-dependent deformation. Loading is applied quasi-statically, without inertial or dynamic rupture effects, and the uniform friction coefficient assigned to all faults does not represent heterogeneous or velocity-dependent frictional behavior observed in nature. Although strike-slip systems are generally scale-invariant, the absolute dimensions of the model domain are considerably smaller than natural fault system, and geometric similarity must be maintained when adapting the workflow. Contact behavior follows the Abaqus implementation and may not translate directly to other finite-element platforms. The workflow does not incorporate pore-fluid pressure or poroelastic coupling.

The above limitations may influence the quantitative accuracy of the simulation results, particularly in terms of absolute stress magnitudes and deformation at depth. However, the primary objective of this workflow is to enable controlled, comparative analysis of deformation patterns under varying convergence angles, rather than to reproduce exact natural conditions. Therefore, the simplifications adopted in the model are not expected to significantly affect the relative differences observed between model scenarios. Future improvements of this workflow could include the incorporation of viscoelastic or rate-dependent rheologies, implementation of dynamic loading conditions, inclusion of heterogeneous or velocity-dependent friction properties, and coupling with pore-fluid pressure effects. Extending the workflow to allow spontaneous fault initiation and propagation would further enhance its applicability to more complex geodynamic systems.

## Ethics statements

This study did not involve human participants, animal experiments, or data collected from social media platforms. No ethical approval was required.

## CRediT author statement

**A.K.:** Methodology; Investigation; Formal analysis; Software; Validation; Visualization; Writing – original draft; Writing – review & editing.

**S.A.A.:** Conceptualization; Supervision; Methodology; Resources; Writing – review & editing.

**R.D.:** Conceptualization; Supervision; Methodology; Resources; Writing – review & editing.

## Declaration of competing interest

The authors declare that they have no known competing financial interests or personal relationships that could have appeared to influence the work reported in this paper.

## Data Availability

The dataset is available on Mendeley Data:https://data.mendeley.com/datasets/fmztbrpnss/1
